# FAHD1 prevents neuronal ferroptosis by modulating R-loop and the cGAS–STING pathway

**DOI:** 10.1515/med-2025-1200

**Published:** 2025-09-24

**Authors:** Bitao Wang, Yubiao Yang, Zhi Zeng, Boyuan Ma, Yuxuan Zhou, Zhenhan Li, Jinyu Chen, Cheng Tang, Jian Hao, Xianhu Zhou

**Affiliations:** Ningbo University Health Science Center, Ningbo, Zhejiang, 315211, China; The Second Affiliated Hospital of Guangzhou Medical University, Guangzhou City, Guangdong Province, 510260, China; Portola High School, 1001 Cadence, Irvine, CA, 92618, USA; The Second Affiliated Hospital of Guangzhou Medical University, No. 250, Changgang East Road, Haizhu District, Guangzhou City, Guangdong Province, 510260, China

**Keywords:** FAHD1, neuronal ferroptosis, oxidative stress, R-loop, cGAS–STING pathway

## Abstract

**Background:**

Ferroptosis, a type of iron-dependent lipid peroxidation-induced neuronal death, has been strongly implicated in the initiation and progression of various neurological disorders, including neurodegenerative diseases and central nervous system (CNS) injuries. Although significant research efforts have been devoted to this area, most available therapeutic strategies remain largely ineffective due to the complex pathophysiology of these conditions. Moreover, the mechanisms underlying neuronal ferroptosis are not yet fully elucidated.

**Methods:**

To investigate the role of FAHD1 in neuronal ferroptosis, bioinformatic analyses and cellular experiments were performed. Immunofluorescence and dot blot analyses were employed to explore the effects of FAHD1 overexpression on R-loop formation. Additionally, western blotting was used to assess alterations in the expression of cGAS–STING pathway-related proteins resulting from FAHD1 overexpression.

**Results:**

Our results demonstrate that FAHD1 expression is significantly downregulated in primary neurons subjected to oxidative stress. Furthermore, ferroptosis appears to be a major contributor to neuronal damage triggered by oxidative stress. Overexpression of FAHD1 significantly reduced reactive oxygen species accumulation and R-loop formation, preserved genomic stability, and suppressed neuronal ferroptosis by inhibiting activation of the cGAS–STING pathway.

**Conclusion:**

FAHD1 is a critical regulator of neuronal ferroptosis and may serve as a potential therapeutic target for the treatment of neurodegenerative diseases and CNS injuries.

## 1 Introduction

Neuron death is a fundamental pathological process in various neurodegenerative diseases and central nervous system (CNS) injuries. The mechanisms contributing to neuronal death include apoptosis, necrosis, autophagy, pyroptosis, ferroptosis, and excitotoxicity [1]. Among these, ferroptosis is one of the significant and has been closely associated with numerous neurodegenerative diseases [2], such as Alzheimer’s disease, Parkinson’s disease, and Huntington’s disease, as well as CNS injuries [3], including spinal cord injury and traumatic brain injury.

Ferroptosis, an iron-dependent form of programmed cell death, is characterized by intracellular iron accumulation and lipid peroxidation [4]. Oxidative stress in the nervous system is closely linked to dysregulation of iron metabolism and lipid peroxidation, making it a critical trigger for ferroptosis [5]. Under conditions of oxidative stress, the body’s antioxidant system may become impaired or imbalanced, reducing its ability to neutralize reactive oxygen species (ROS). This deficiency leads to excessive ROS accumulation, triggering harmful processes such as lipid peroxidation and disruptions in iron metabolism, which ultimately result in cellular ferroptosis. Studies have shown that ROS inhibitors, such as N-acetylcysteine [6], and iron chelators, such as ferrostatin-1 [7], effectively reverse neuronal ferroptosis. Inhibiting ferroptosis preserves a substantial number of functional neurons and oligodendrocytes, significantly mitigating functional loss [8]. The complexity of the etiology and pathology of neurological disorders, coupled with the presence of the blood–brain barrier, render conventional pharmacological therapies ineffective in many cases. At present, in excess of 95% of patients are without an efficacious treatment option [9]. Thus, reducing neuronal ferroptosis is crucial for maintaining nervous system function. However, the full mechanisms underlying ferroptosis remain incompletely elucidated.

Accumulation of R-loops has been implicated in several neurological disorders, including ataxia, neuromuscular diseases, and spinal muscular atrophy, among others [10]. R-loops are triple-stranded structures consisting of RNA–DNA hybrids and unpaired single-stranded DNA, typically formed during transcription. These structures play a crucial role in gene regulation and maintaining genomic stability [11]. Oxidative stress can lead to the excessive production of ROS, resulting in abnormal R-loop accumulation and genomic instability [12], which subsequently activates the cGAS–STING-mediated inflammatory response [13]. Research indicates that reducing R-loop formation by alleviating oxidative stress is essential in ensuring neuronal health and homeostasis [14]. Nevertheless, our understanding of R-loop functions in the nervous system remains limited.

FAHD1, a member of the FAH protein family, catalyzes the conversion of fumarate to malate, a key reaction in the tricarboxylic acid (TCA) cycle [15]. Previous studies have demonstrated that FAHD1 is crucial for mitochondrial function. FAHD1 deficiency disrupts TCA cycle flux, impairs cell proliferation, inhibits mitochondrial energy metabolism, and ultimately leads to premature cellular senescence [16]. Additionally, FAHD1 plays an essential role in regulating cellular ROS levels, as overexpression of FAHD1 has been shown to shift cellular metabolism toward glycolysis and reduce ROS accumulation [17]. Furthermore, FAHD1 has been identified as a regulator of serotonin levels, underscoring the intricate relationship between neuronal metabolism and neurotransmitter signaling [18]. Transcriptome sequencing has revealed a significant reduction in FAHD1 expression in mouse hippocampal cell line (HT22) during oxidative stress. However, the precise role of FAHD1 in oxidative stress-induced neuronal ferroptosis remains to be defined.

This study aims to investigate the role of FAHD1 in neuronal ferroptosis and to elucidate its underlying mechanisms. Our results suggest that FAHD1 is a crucial regulator of neuronal ferroptosis. Overexpression of FAHD1 reduces oxidative stress-induced ROS production, thereby attenuating R-loop formation, enhancing genomic stability, and preventing neuronal ferroptosis by inhibiting the cGAS–STING signaling pathway. In conclusion, this study demonstrates that FAHD1 plays a central role in the pathogenesis of neuronal ferroptosis and suggests that FAHD1 may represent a potential therapeutic target for neurological diseases.

## 2 Materials and methods

### 2.1 Chemicals and cell

Enhanced Cell Counting Kit-8 (CCK8; Cat. No. C0042), LDH Assay Kit with WST-8 (Cat. No. C0018S), ROS (Cat. No. S0033S), and RNase III (Cat. No. R7086S) were obtained from Shanghai Beyotime Biotechnology (Shanghai, China). 5,6-Dimethylxanthenone-4-acetic acid (DMXAA) (Cat. No. HY-10964, MedChemExpress, USA), Ferrostatin-1 (Fer-1; Cat. No. SML0583, Sigma-Aldrich, USA), RU.521 (MedChemExpress, USA), GenElute™ (Cat. No. G1N70, Sigma-Aldrich, USA), malondialdehyde (MDA; Cat. No, A003-2), and glutathione (GSH; Cat. No. A006-2-1) were acquired from Jiancheng Bioengineering Institute (Nanjing, China). Hydrogen peroxide (H_2_O_2_; Cat. No. H433857) was obtained from Shanghai Aladdin Biochemical Technology Co., Ltd (Shanghai, China). Fetal bovine serum (Cat. No. 10270106) and Dulbecco's modified eagle medium (DMEM) (Cat. No. C11995500BT) were sourced from Gibco. FastPure^®^ Cell Total RNA Isolation Kit V2, PrimeScriptTM RT reagent kit, and SYBR^®^ Premix Ex TaTM were purchased from Vazyme (Nanjing, China). Plasmids were purchased from Ruibo Biotechnology Co (Guangzhou, China). Lipofectamine 3000 from Invitrogen, USA. The vector was generated by Obio Technology (Shanghai, China). HEK 293T cell line was purchased from OriCell Biotechnology Ltd (Guangzhou, China).

### 2.2 Animals

Pregnant C57 mice were purchased from Zhuhai Beston Biotechnology Co. All animal care and experimental protocols were reviewed and approved by the Experimental Animal Welfare Ethics Review Committee of the Second Affiliated Hospital of Guangzhou Medical University (Approval number: A2023-049).

### 2.3 Primary neuron isolation and culture

Pregnant C57 mice were euthanized, immersed in ice-cold ethanol for 30 s, and dissected to extract the fetal brains, which were rinsed in cold phosphate-buffered saline (PBS). A microscope was used to remove the skull, blood, and meninges from the fetal brains. The cortex was isolated and transferred to a papain solution, incubated at 37°C for 20–30 min to facilitate cell dissociation. The tissue was triturated using a cell strainer or pipette, resulting in a single-cell suspension. The suspension was centrifuged to remove the enzyme solution, then resuspended in neuron-specific growth medium and seeded into precoated dishes. The cultures were maintained in a 37°C, 5% CO_2_ incubator with periodic medium changes. All experiments were performed in the surgical laboratory of the Second Affiliated Hospital of Guangzhou Medical University.

### 2.4 Establishment of an *in vitro* oxidative stress model

Oxidative stress was induced by incubating primary neurons with 100 μM H_2_O_2_ in neuronal culture medium for 12 h, following established protocols [19]. After incubation, neurons were collected for analyses, including quantitative reverse transcription PCR (qRT-PCR), western blotting, immunofluorescence (IF), and dot blot assays.

### 2.5 Lentivirus infection and cell transfection

A lentivirus infection was conducted to knock down RNase H1 (shRNH1) or over-express FAHD1 (OE) in neurons. The plasmid was cloned into a lentivirus vector and transfected into HEK-293T cells. The virus was used to infect neurons. Around 1 × 10⁵ neurons were seeded into each well of a six-well plate for transfection. Lipofectamine 3000 was used to transfect plasmids into cells. Western blotting was used to determine transfection efficiency. An empty pSLenti-EF1-P2A-Puro-CMV-MCS-3xFLAG-WPRE vector was used as a control. The following shRNA sequences were used to target RNH1: AGGCAAAGAAATCTATTAGT [20].

### 2.6 CCK8 assay

Neurons viability was measured using the CCK8 assay. Neurons were seeded in 96-well plates at a density of 5 × 10^3^ cells/well. After treatment, CCK8 solution was added to per well and incubated for 2 h. Then the absorbance value was measured at 450 nm, and the relative viability of cells was calculated with the blank control group.

### 2.7 Assessment for GSH level, MDA level, and LDH release

According to the manufacturer’s guidelines, GSH and MDA kits were used to detect the levels of GSH and MDA. In addition, LDH release in the cell supernatant was measured using a LDH release assay kit.

### 2.8 Detection of intracellular ROS

ROS assay kit was employed to detect the total level of intracellular ROS. After different treatments, neurons were washed with PBS three times, and then incubated with serum-free DMEM medium containing dichlorodihydrofluorescein diacetate (DCFH-DA) at 37°C in the dark for 2 h, and then discarded the medium. After washing with PBS three times, the outcomes were acquired using the Olympus inverted fluorescence microscope.

### 2.9 qRT-PCR

Total RNA was extracted from the neurons using the FastPure^®^ Cell Total RNA Isolation Kit V2, in accordance with the manufacturer's instructions. RNA concentrations were assessed using a NanoDrop spectrophotometer. Subsequently, RNA was used to synthesize cDNA through reverse transcription with the PrimeScriptTM RT reagent Kit. Gene expression analysis was performed on a QuantGene 9600 Real-Time PCR System using SYBR^®^ Premix Ex TaTM. Quantification for target gene expression was performed using the 2^−ΔΔCt^ method and β-actin was used as normalization control. All primers are given in Table S1.

### 2.10 Western blot

For western blot analysis, cells were harvested and lysed with RIPA buffer for 30 min on ice. The samples were subjected to SDS-PAGE and transferred to PVDF membranes via a wet transfer technique. The membranes were blocked with 5% non-fat milk for 2 h, then incubated overnight at 4°C with the appropriate primary antibodies. After three 10 min washes in TBST, membranes were incubated with secondary antibodies for 1 h at room temperature. Protein bands were detected using Immobilon Western horseradish peroxidase (HRP) mix reagent (Millipore). Antibodies used are listed in Table S2.

### 2.11 IF

Neurons were seeded on glass coverslips in six-well plates and subsequent implementation of various treatments. Subsequently, the neurons were rinsed three times with PBS, fixed with 4% formaldehyde, and permeabilized using 0.5% Triton X-100. Subsequently, the cells were blocked with 5% bovine serum albumin for 1 h. Next, the cells were incubated overnight at 4°C with a primary antibody, followed by incubation with a fluorescein isothiocyanate-conjugated goat secondary antibody for 1 h in the dark. Finally, the nuclei were stained with 4′,6-diamidino-2-phenylindole and visualized using an Olympus inverted fluorescence microscope. The average fluorescence intensity was quantified using ImageJ.

### 2.12 Dot blot analysis

Genomic DNA was extracted using the GenElute™ kit. The isolated gDNA was treated with RNase III for 2 h at 37°C. The enzyme was inactivated at 65°C for 20 min, and then the samples were divided. The control samples were digested with 10 U RNaseH1 for 16 h at 37°C. The DNA was spotted onto a nylon membrane using a dot-blot apparatus. The DNA was cross-linked to the membrane using ultraviolet light, and a 5% skimmed milk to block it. The membrane was incubated at 4°C overnight with S9.6 or dsDNA. The antibody was washed for 10 min in TBST, then HRP-conjugated secondary antibodies were applied for 1 h at room temperature. The intensity of the signals was quantified using ImageJ, and the ratios between the S9.6 and dsDNA signals were calculated to ascertain the global levels of R-loops.

### 2.13 Statistical analysis

Statistical analyses were performed using Graphpad Prism 10.2 software and are presented as mean ± SEM. Differences between variables were considered statistically significant at *p* < 0.05, **p < 0.01, ***p < 0.001, ****p < 0.0001, and ns, not significant.


**Ethical approval:** All animal care and experimental protocols were reviewed and approved by the Experimental Animal Welfare Ethics Review Committee of the Second Affiliated Hospital of Guangzhou Medical University (Approval number: A2023-049).

## 3 Result

### 3.1 Expression of FAHD1 is diminished during oxidative stress in neurons

**Figure 1:**
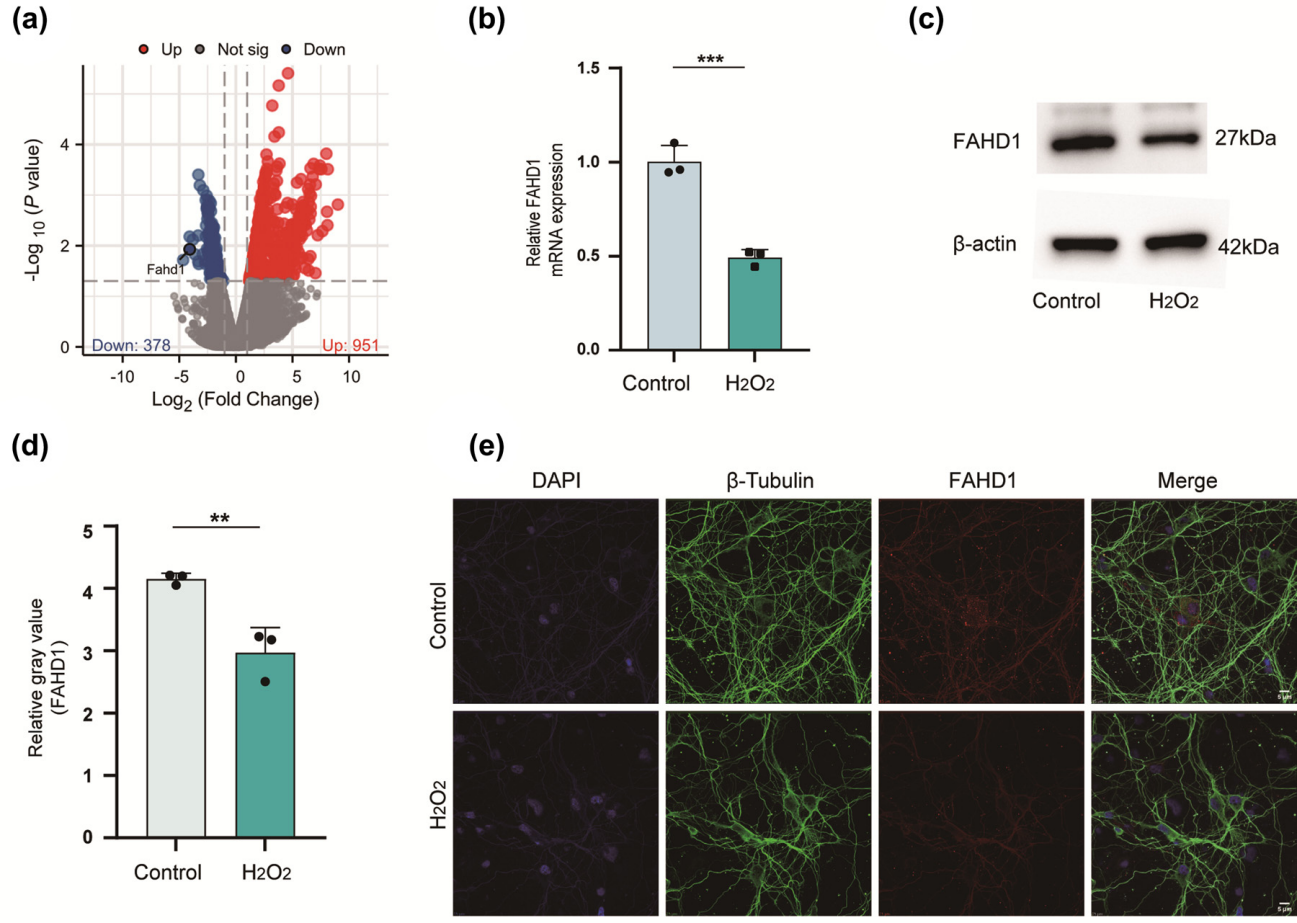
FAHD1 expression in neurons under oxidative stress. Volcano plot of differentially regulated genes under oxidative stress (a). qRT-PCR evaluated FAHD1 expression levels after 100 µM H_2_O_2_ treatment (b). Western blot and semi-quantitative analysis of FAHD1 expression (c) and (d). IF staining analysis of FAHD1, scale bar: 5 μm (e) (*n* = 3, one sample *t*-test, **p < 0.01 and ***p < 0.001 represent comparison with control group).

The HT22 is a frequently utilized mouse hippocampal neuronal cell line that exhibits characteristic neuronal morphological features. It has been extensively employed in research pertaining to neurodegenerative diseases, oxidative stress, and signaling pathways. The objective of this study was to analyze specific molecules associated with neuronal oxidative stress. To this end, we performed a comprehensive analysis of the HT22’s transcriptome, with the aim of identifying genes associated with oxidative stress. As demonstrated in the volcano plot, among the differentially expressed genes, the expression of FAHD1 was significantly reduced (Figure 1a). To further substantiate this finding, RT-qPCR (Figure 1b), western blotting (Figure 1c and d), and IF (Figure 1e) consistently demonstrated a significant reduction in FAHD1 expression in the experimental group compared to the control group, in alignment with the bioinformatics analysis. Previous research has demonstrated that FAHD1 plays a role in regulating ROS production, leading us to hypothesize that FAHD1 is a key factor in protecting cells against oxidative stress damage.

### 3.2 Ferroptosis mediates H_2_O_2_-induced injury in neurons

**Figure 2:**
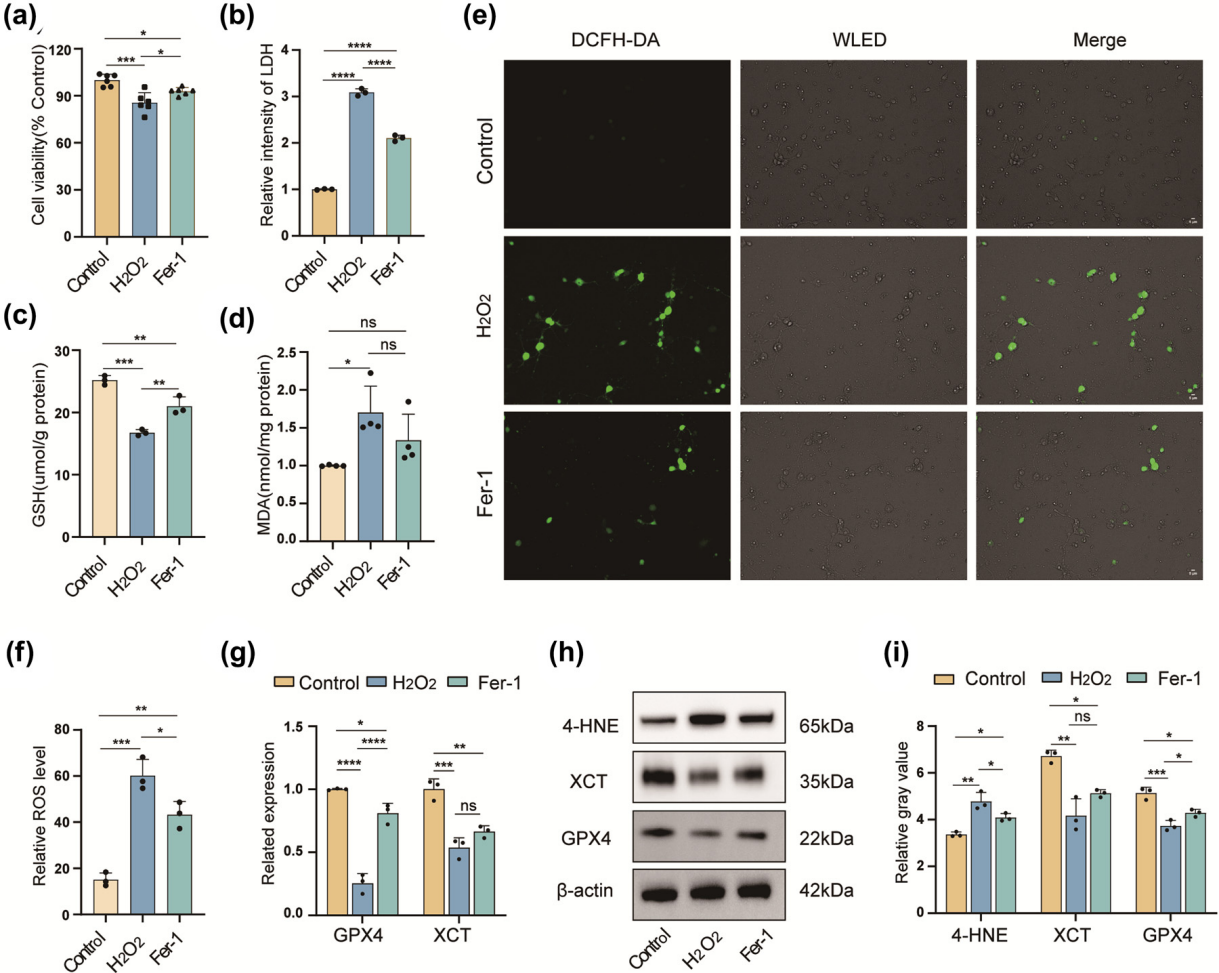
Ferroptosis causes H_2_O_2_-induced neuronal damage. Cell viability evaluated using the CCK-8 assay (a) (*n* = 6, one-way ANOVA, *p < 0.05, ***p < 0.001). An LDH assay (b) assessed cellular damage. GSH (c) and MDA (d) levels quantified. ROS production was evaluated using the DCFH-DA assay, scale bar 5 µm (e) and (f). WELD: White light emitting diode. qRT-PCR evaluated GPX4 and XCT expression levels (g). Ferroptosis-related proteins, such as 4-HNE, GPX4, and XCT were detected by western blotting (h) and (i) (*n* = 3, one-way ANOVA, *p < 0.05, **p < 0.01, ***p < 0.001, and ns, not significant).

The contribution of ferroptosis to H_2_O_2_-induced neuronal damage is illustrated in Figure 2. Treatment with H_2_O_2_ caused a significant decrease in neuronal survival (Figure 2a and b) and GSH levels (Figure 2c). GSH, a critical intracellular antioxidant, prevents lipid peroxidation by activating glutathione peroxidase 4 (GPX4), thereby inhibiting ferroptosis. Conversely, levels of MDA, a marker of lipid peroxidation, and ROS were significantly elevated (Figure 2d–f). Furthermore, qRT-PCR analysis demonstrated reduced expressions of SLC7A11 (also known as XCT) and GPX4 in neurons exposed to H_2_O_2_ (Figure 2g). Western blotting confirmed a significant reduction in ferroptosis-related proteins (XCT and GPX4) and a marked increase in 4-hydroxynonenal (4-HNE) levels (Figure 2h and i). Importantly, the neuronal damage induced by H_2_O_2_ was partially reversed by 1 µM Fer-1 [21], a ferroptosis inhibitor (Figure 2). These findings suggest that ferroptosis plays a key role in H_2_O_2_-induced neuronal death.

### 3.3 Overexpression of FAHD1 reduces the formation of R-loop and DNA damage in neurons

**Figure 3:**
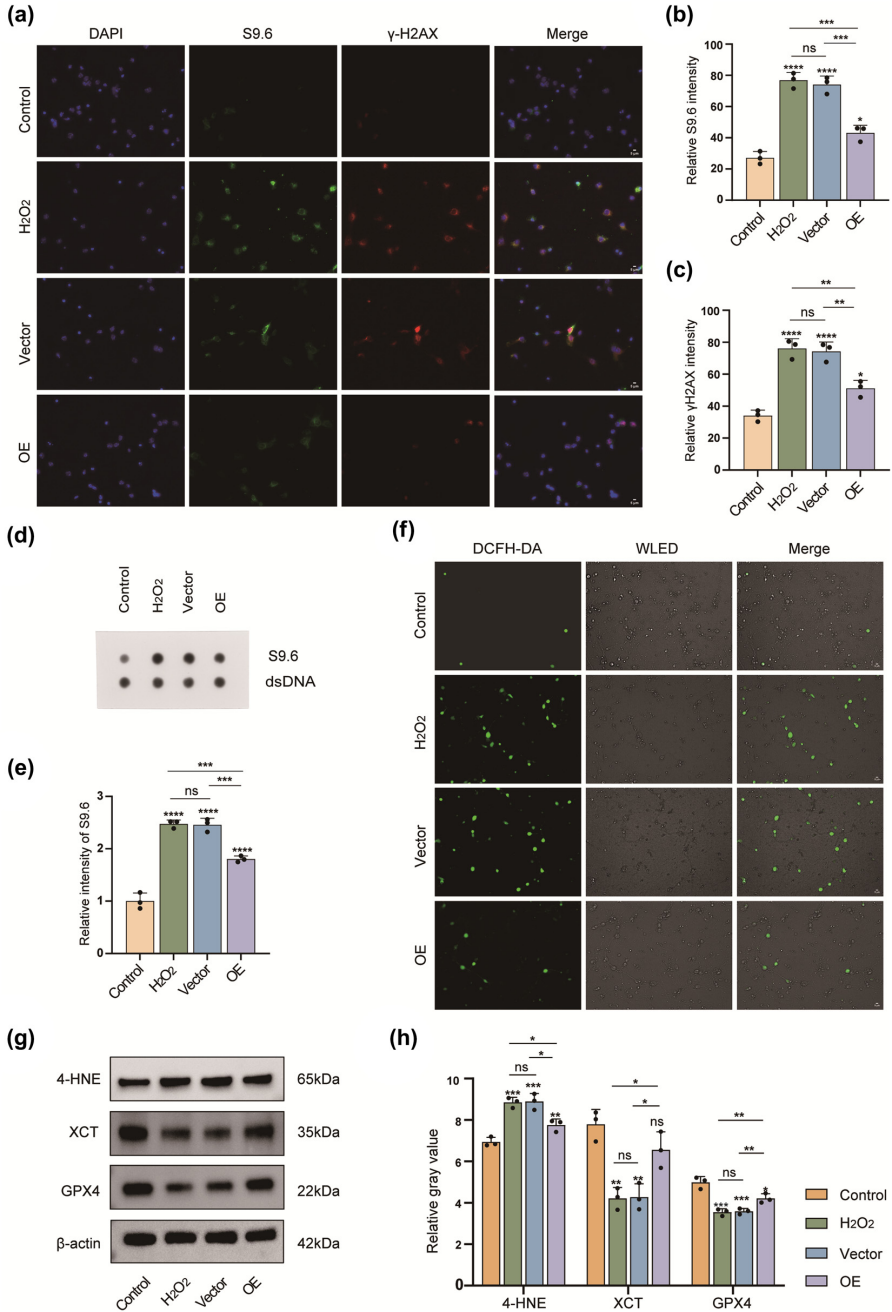
Overexpression of FAHD1 reduces R-loop formation and DNA injury, while also mitigating ferroptosis. IF was performed to detect S9.6 and γ-H2AX expression, followed by semi-quantitative analysis, scale bar: 5 μm (a)–(c). Dot blot analysis and semi-quantitative analysis were conducted to assess R-loops using an antibody against S9.6, with dsDNA as an internal loading control (d) and (e). DCFH-DA assay employed to evaluate the production of ROS, scale bar: 5 μm (f). 4-HNE, XCT, and GPX4 expression levels were detected by western blotting and semi-quantitative analysis (g) and (h) (*n* = 3, one-way ANOVA, *p* < 0.05, **p < 0.01, ***p < 0.001, ****p < 0.0001, and ns, not significant).

ROS generated during oxidative stress can promote the accumulation of abnormal R-loops and induce DNA damage. To determine whether FAHD1 mitigates R-loop formation and reduces DNA damage by inhibiting ROS, we performed IF staining. The results showed elevated levels of the R-loop marker S9.6 [22] and the DNA damage marker phosphorylated H2AX (γ-H2AX) [23] in neurons following H_2_O_2_ treatment. However, FAHD1 overexpression significantly attenuated these effects (Figure 3a–c). The successful transfection of FAHD1 was confirmed by western blot analysis (Figure S1a). Moreover, dot blot analysis confirmed that FAHD1 overexpression resulted in a decrease in S9.6 levels (Figure 3d and e). Furthermore, ROS levels were significantly elevated following H_2_O_2_ treatment, but markedly reduced by FAHD1 overexpression (Figure 3f). These findings suggest that FAHD1 overexpression decreases R-loop formation, thereby reducing DNA damage and promoting genomic stability in neurons.

### 3.4 Overexpression of FAHD1 inhibits ferroptosis in neurons

As previously described, overexpression of FAHD1 inhibited neuronal ROS production. In addition, we observed that FAHD1 overexpression reversed the H_2_O_2_-induced elevation of MDA levels and the reduction of GSH levels in neurons (Figure S2a–c). Furthermore, western blot analysis demonstrated that H_2_O_2_ treatment resulted in a downregulation of GPX4 and XCT expression, accompanied by an upregulation of 4-HNE levels. In contrast, FAHD1 overexpression reduced 4-HNE levels while upregulating GPX4 and XCT expression (Figure 3g and h). These results suggest that FAHD1 overexpression mitigates oxidative stress-induced neuronal ferroptosis.

### 3.5 FAHD1 attenuates oxidative stress-induced neuronal ferroptosis by inhibiting cGAS–STING pathway

**Figure 4:**
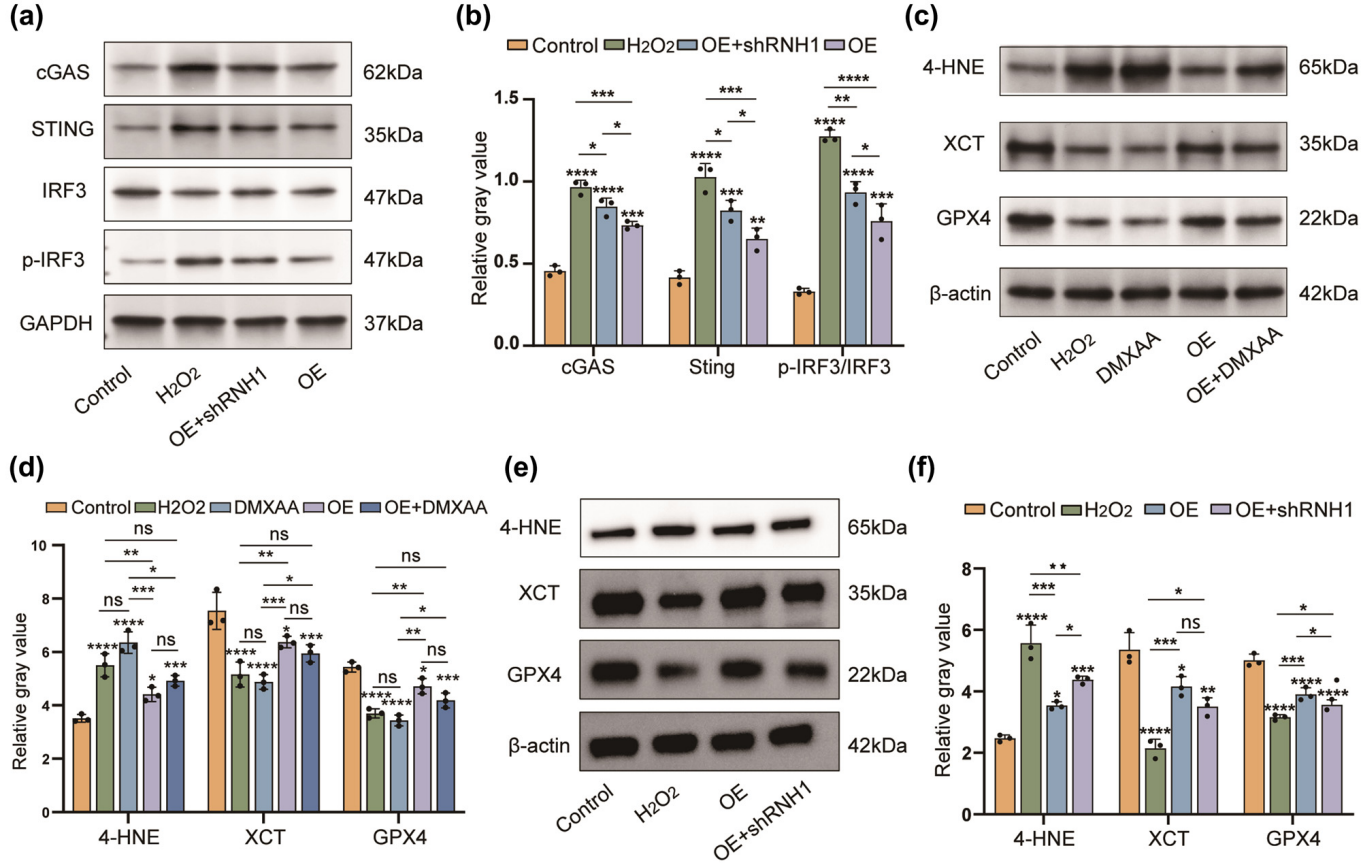
FAHD1 attenuates neuronal ferroptosis by inhibiting the cGAS–STING pathway through reducing aberrant R-loop formation. Western blotting and semi-quantitative analysis were performed to assess the expression of cGAS–STING pathway-related proteins, including cGAS, STING, p-IRF3, and IRF3 (a) and (b). DMXAA: STING pathway agonist. After different treatments, the expression levels of ferroptosis-associated proteins 4-HNE, XCT, and GPX4 were detected by western blotting and quantified using ImageJ software (c)–(f) (*n* = 3, one-way ANOVA, *p* < 0.05, **p < 0.01, ***p < 0.001, ****p < 0.0001, and ns, not significant).

Previous studies have identified the cGAS–STING pathway as a crucial mechanism in innate immune regulation, responsible for detecting abnormal intracellular DNA [24]. The cGAS–STING pathway has been identified as a potential contributor to a range of neurological disorders, including neurodegenerative diseases [25] and CNS injury [26]. To explore whether FAHD1 is potentially associated with the cGAS–STING pathway, western blot analysis demonstrated that H_2_O_2_ stimulation activates this pathway in neurons, increasing the expression of cGAS, STING, and phosphorylated IRF3. Conversely, FAHD1 overexpression diminished the expression of these proteins. Interestingly, the knockdown of RNase H1(RNH1), which degrades the RNA portion of the R-loop to restore DNA’s double-stranded structure [27], rendered FAHD1’s effects null (Figure 4a and b). The knockdown effect of RNH1 was confirmed through western blot analysis (Figure S1b). To verify that FAHD1 mitigates ferroptosis through the inhibition of the cGAS–STING pathway, western blot analysis revealed that FAHD1 overexpression resulted in enhanced GPX4 and XCT expression and attenuated 4-HNE levels. However, treatment with the STING agonist DMXAA [28] reversed these changes in protein expression (Figure 4c and d). Additionally, the decrease in neuronal survival induced by H2O2 was partially rescued by 1 μM RU.521 [29], a cGAS–STING pathway inhibitor (Figure S3a and b). CCK-8 assays confirmed that DMXAA exhibited negligible toxicity to neurons at concentrations below 50 µg/mL (Figure S3c). Similarly, the knockdown of RNH1 reversed the alterations in FAHD1-mediated expression of ferroptosis-related proteins (Figure 4e and f). The aforementioned results indicate that FAHD1 diminishes the activation of the cGAS–STING pathway by limiting the aberrant accumulation of R loops, which ultimately results in a reduction of neuronal ferroptosis.

## 4 Discussion

Ferroptosis represents a form of cell death characterized by iron accumulation and lipid peroxidation, both of which are prevalent pathophysiological features associated with neuronal oxidative stress [30]. This form of cell death is central to various neurological disorders [31]. In neurodegenerative diseases and CNS injuries, oxidative stress results in elevated levels of ROS and inflammatory factors. If these harmful compounds are not effectively scavenged or regulated, they propagate and form more complex derivatives, exacerbating neuronal damage. Previous studies have shown that inhibiting microglial ferroptosis significantly mitigates neurodegenerative changes [32]. Additionally, zinc has been found to attenuate ferroptosis and facilitate functional recovery following contusion spinal cord injury through the activation of the Nrf2/GPX4 defense pathway [33]. Therefore, early inhibition of neuronal ferroptosis is crucial to preserve the functional stability of the nervous system.

Previous studies have shown that the abnormal accumulation of R-loops is a common phenomenon in various human diseases, including neurological disorders and cancers [34]. R-loops are widely found in cells and are normally regulated dynamically under physiological conditions by precise formation and elimination mechanisms. However, abnormal R-loop accumulation leads to genomic instability, which can trigger various pathological conditions. Previous research has indicated that the inhibition of the aberrant accumulation of ROS and R-loop, which are generated by cellular oxidative stress, plays a pivotal role in maintaining neuronal cell health and homeostasis.Therefore, activating antioxidant mechanisms is essential to limit R-loop accumulation in the nervous system and maintain genome stability.

The growing body of evidence confirms that ROS accumulation stimulates the formation of aberrant R-loops, closely associated with the activation of the cGAS–STING pathway. Our experimental findings indicate that FAHD1 overexpression reduces neuronal ROS levels, decreases R-loop formation, and inhibits neuronal ferroptosis by suppressing the cGAS–STING pathway. Previous studies have also noted FAHD1 expression in both mouse and human models, highlighting its regulatory roles in energy metabolism, cellular function, and aging. In this study, we further demonstrate FAHD1’s critical role in mediating the pathological processes linked to neuronal ferroptosis.

Although this study has certain limitations, it offers valuable insights. Our findings indicate that FAHD1 reduces R-loop formation and mitigates neuronal ferroptosis through the cGAS–STING pathway. However, the precise mechanisms involved remain to be fully elucidated. Furthermore, it is unclear whether FAHD1 exerts additional functions, and our understanding of its role is still in the early stages of development. There are also substantial gaps in our knowledge regarding the mechanisms of ferroptosis in neurodegenerative diseases and CNS injury, warranting further investigation. In summary, addressing neuronal ferroptosis and preserving the functional stability of the nervous system continue to present significant challenges.

## Supplementary Material

Supplementary material
