# Supplementary material for: FAHD1 prevents neuronal ferroptosis by modulating R-loop and the cGAS–STING pathway
Source: Open Med (Wars). 2025 Sep 24;20(1):20251200. doi: 10.1515/med-2025-1200 (PMC12487764; doi:10.1515/med-2025-1200)
Supplement: Supplementary material [file med-2025-1200-sm.pdf]

Supplementary material

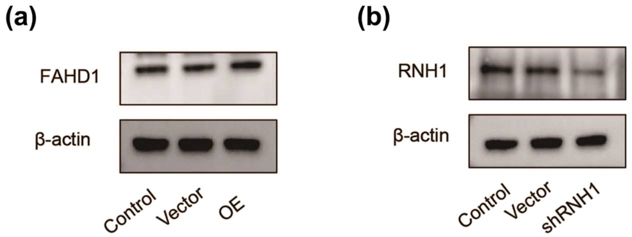

Figure S1: Overexpression of FAHD1 or knockdown of RNH1 in neurons. Overexpression of FAHD1 (a) knockdown of RNH1 (b).

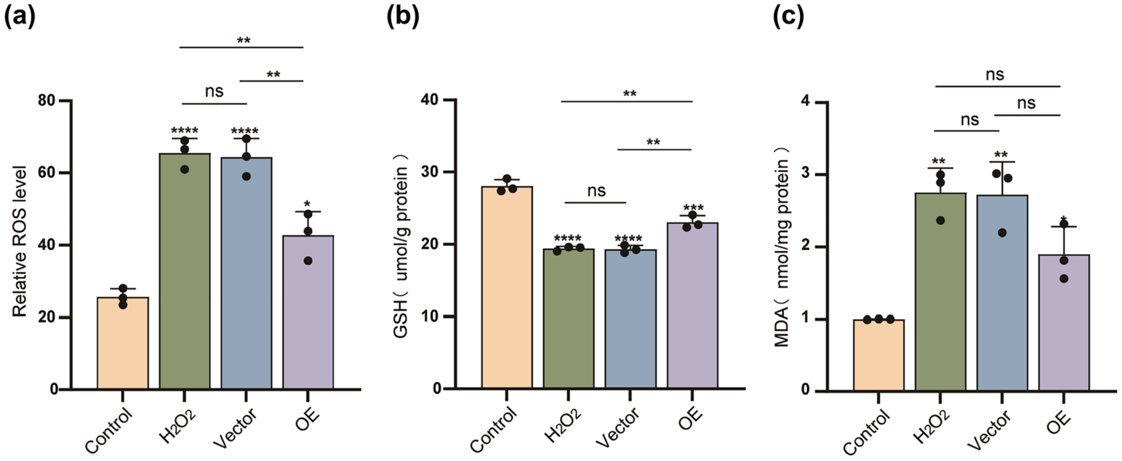

Figure S2: Overexpression of FAHD1 reduces ROS, mitigating oxidative stress-induced ferroptosis. Assay for evaluating ROS production (a). GSH levels were quantified (b). MDA levels were quantified (c). (n = 3, one sample t test, *P < 0.05, **p < 0.01, ***p < 0.001 and ****p < 0.0001 represent comparison with Control group).

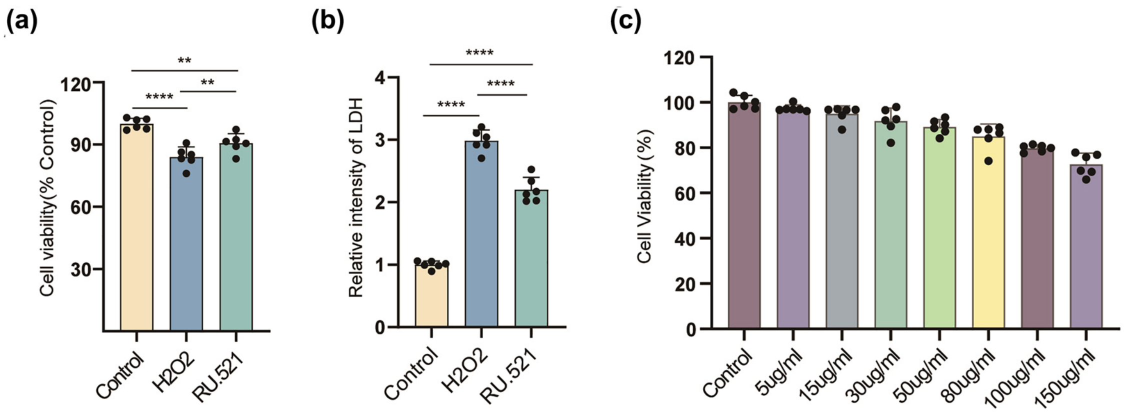

Figure S3: Cell viability was assessed using the CCK-8 assay (a), and cellular damage was evaluated through the LDH assay (b). The effect of varying concentrations of DMXAA on neuronal viability was examined (c). (n = 6, one-way ANOVA, $^{**}P < 0.01$, $^{****}P < 0.0001$).

Table S1: Primers used for qRT-PCR

Abbreviation	Sequences	
	Forward	Reverse
β-actin	TGAGCTGCGTTTTACACCCT	GCCTTCACCGTTCCAGTTTT
FAHD1	TCTGCGTGGGGAGGAACTAT	CGGCTTCAGGAAAAGCACAG
GPX4	CGCCAAAGTCCTAGGAAACG	AAGGTTCAGGAATGGGCTCC
XCT	TCCGAGGAGCAAGAGGAGTAAT	ATCACTGTTCGGTCGTGACTT

Table S2: Antibodies

Name	Dilution	Species	Manufacture	Cat, No.	Application
FAHD1	1:1,000	Rabbit	Proteintech	26233-1-AP	WB
	1:200				IF
γ-H2AX	1:250	Rabbit	abcam	ab81299	IF
4-HNE	1:1,000	Rabbit	abcaam	ab46545	WB
XCT	1:1,000	Rabbit	abcam	ab307601	WB
Gpx4	1:5,000	Rabbit	abcam	ab125066	WB
GAPDH	1:2,000	Mouse	abcam	ab8245	Wb
S9.6	1:50	Rabbit	MERCK	MABE1095	IF
	0.2 ug/ml				Dot Blot
dsDNA	1:1,000	Mouse	MERCK	ZMS1047	Dot Blot
cGAS	1:1,000	Rabbit	Cell Signaling Technology	#31659	Wb
STING	1:1,000	Rabbit	Cell Signaling Technology	#13647	Wb
IRF3	1:1,000	Rabbit	Cell Signaling Technology	#4302	Wb
P-IRF3	1:1,000	Rabbit	Cell Signaling Technology	#29047	Wb
β-tubulin	1:50	Rabbit	Cell Signaling Technology	#2142	IF
β-actin	1:30,000	Mouse	Proteintech	66009-1-Ig	WB
Goat anti Mouse IgG (H+L)	1:3,000	Goat	ZSGB-BIO	ZB-5305	WB
Goat Anti Rabbit IgG (H+L) HRP	1:5,000	Goat	Affinity	#S0001	WB

WB: Western blot, IF: Immunofluorescence.
